# Influenza Vaccine Effectiveness in the Tropics: Moderate Protection in a Case Test-Negative Analysis of a Hospital-Based Surveillance Population in Bangkok between August 2009 and January 2013

**DOI:** 10.1371/journal.pone.0134318

**Published:** 2015-08-12

**Authors:** Jens W. Levy, Sriluck Simasathien, Veerachai Watanaveeradej, Piraya Bhoomiboonchoo, Stefan Fernandez, Richard G. Jarman, Chonticha Klungthong, Robert V. Gibbons, Phirangkool Kerdpanich, Danaband Piboonbanakit, Tundorn Chirabandhu, In-Kyu Yoon

**Affiliations:** 1 Department of Virology, Armed Forces Research Institute of Medical Sciences, Bangkok, Thailand; 2 Phramongkutklao Hospital, Bangkok, Thailand; 3 Viral Disease Branch, Walter Reed Army Institute of Research, Silver Spring, Maryland, United States of America; Georgia State University, UNITED STATES

## Abstract

Influenza in the tropics occurs year round with peaks that correspond variably to temperate regions. However, data on influenza vaccine effectiveness (VE) in the tropics is sparse. We report on the effectiveness of influenza vaccine to prevent medically attended laboratory confirmed influenza from sentinel surveillance conducted at a Thai military medical facility in Bangkok, Thailand from August 2009 to January 2013. Patients ≥6 months old presenting with influenza-like illness underwent combined nasal/throat swabs which were tested by influenza RT-PCR. A case test-negative study design was used to evaluate VE. Of 2999 samples available for analysis,1059 (35.3%) were PCR-positive (cases) and 1940 (64.6%) were PCR-negative (test-negative controls). Five hundred and seven (16.9%) of these patients reported being vaccinated within the previous 12 months. Periods of high and low influenza activity were defined based on publicly available Thai Ministry of Public Health data. Overall VE adjusted for age and epiweek was found to be 50.1% (95%CI: 35.0, 61.9%). The May to April adjusted VE for year 2010, 2011 and 2012 was 57.7% (95%CI: 33.7, 73.8%), 57.1% (95% CI: 35.2, 68.3%) and 37.6% (95% CI: 3.5, 62.9%).During high influenza activity in years with the same vaccine formulation, the adjusted VE was 54.9% (95%CI: 38.9, 66.9%). VE appeared to be much higher during high versus low influenza activity periods. The adjusted point estimate for VE was highest in the 18–49 year age group (76.6%) followed by 6–23 months (58.1%) and 2–17 years (52.5%). Adjusted estimates were not done for those ≥50 years of age due to small numbers. VE in patients with underlying disease was 75.5% compared to 48.0% in those without. Our findings demonstrate moderate protection by influenza vaccination and support the utility of influenza vaccination in the tropics including in very young children and those with underlying disease.

## Introduction

East and Southeast Asian countries occupy a unique position in the global ecology of influenza[1]. Influenza in these countries has a sustained background transmission activity with peaks that variably correspond to the temperate regions of the Northern and Southern Hemispheres [2]. Year round influenza provides a reservoir from which new influenza virus strains and lineages may emerge that subsequently seed virus circulation in the temperate regions [3]. Globally, influenza is recognized as a significant cause of morbidity and mortality (WHO).[4]. However, only recently has the considerable impact of influenza virus infections in Southeast Asia been elucidated [5, 6].This recognition, together with the advent of global concerns over avian influenza and the recent H1N1 influenza pandemic (A(H1N1)pdm09), has spurred an increase in vaccination programs supported by many Southeast Asian governments [7]. However, data on influenza vaccine effectiveness (VE) in this region remain sparse.

The Thailand Ministry of Public Health (MOPH) has offered influenza vaccination for free to health care workers since 2004 [7]. Since then, the vaccination recommendations have evolved. Beginning in 2009, the MOPH has recommended influenza vaccination for pregnant women, children between 6 months and 2 years old, adults age 65 years and older, persons with underlying medical conditions, institutionalized mentally ill individuals, and persons over 100 kg. In Thailand, influenza virus circulates year round, with a peak approximately corresponding to that of the Southern Hemisphere, typically after the beginning of the rainy season in June or July[8]. A smaller second peak frequently occurs between October and February but not every year. While both Northern and Southern Hemisphere vaccines may be used, in Thailand, the Southern Hemisphere vaccine predominates and public sector immunization usually occurs between May and July (Piyarat Suntarratiwong by personal communication). Virtually all influenza vaccines used in Thailand are of the inactivated type.

Monitoring of VE to evaluate the performance of vaccinations under field conditions is important to inform vaccination policy in Thailand and the region as well as maintain public confidence in influenza vaccination programs. VE has been well studied in temperate regions and in the context of distinct influenza seasons. VE has been shown to vary due to antigenic mismatch between the vaccine and circulating strains [9]. Effectiveness may also change with antigenic drift over the course of a season. In the tropics, these vagaries are compounded by year round circulation unconstrained by season. In Thailand and Indonesia, the circulating strains have been shown to only partially match the recommended vaccine strains with some circulating strains preceding the vaccination strains by years [8, 10]. Therefore, it is uncertain whether influenza vaccine effectiveness in the tropics is similar to that observed in temperate regions. Comparing the annual southern hemisphere VE in the tropical setting with other countries where influenza reporting runs between May and the following April is complicated by the variable seasonality of influenza in the tropics.

In the current study, we report on the effectiveness of influenza vaccine to prevent medically attended, laboratory confirmed influenza identified from sentinel surveillance established at Phramongkutklao Hospital (PMK), a military medical facility in Bangkok, Thailand, serving active and retired Royal Thai Army (RTA) military personnel, their families, and other civilians, shortly after the beginning of the 2009 influenza pandemic. The first A(H1N1)pdm09 case in Thailand occurred in June 2009 and the surveillance program at PMK began in August 2009. In late 2009, the Thai government received 2 million doses of monovalent A(H1N1)pdm09 vaccine. Beginning in January 2010, about 5,000 doses of this monovalent pandemic vaccine were provided to active duty RTA soldiers. In July 2010, a seasonal trivalent vaccine that included A(H1N1)pdm09 began to be dispensed at PMK to high risk groups. This trivalent vaccine was also available in Thailand for a fee to all individuals as early as April 2010. Prior to this, any civilian receiving vaccination at PMK was administered trivalent vaccine containing the prior seasonal A(H1N1) strain. Our study is unique among reports of influenza vaccine effectiveness as it includes VE estimates year round in a tropical country in all ages, across several periods of both high and low influenza activity.

## Methods

### Study Population

Sentinel surveillance for influenza-like illness (ILI) was established as part of the Armed Forces Research Institute of Medical Sciences (AFRIMS) influenza surveillance program in August 2009 at PMK in Bangkok, Thailand. The source population included adults (military and civilian) and children ≥6 months old in Bangkok.

### Study Procedures

Study staff recruited patients ≥6 months of age presenting with ILI defined as having fever (>38°C) and cough or sore throat from outpatient and inpatient departments at PMK. Eligible patients had fever onset within 3 days prior to presentation for outpatients and 5 days for inpatients. Study staff obtained demographic and clinical information from enrolled subjects and/or their parents (if applicable). This information included underlying medical conditions and whether or not the subject had received influenza vaccine within the previous 12 months along with the date of vaccination (if known). A nasal swab was obtained to perform a rapid influenza test (QuickVue) to inform clinical care. Finally, a set of combined nasal and throat swabs were placed in viral transport media and stored at -20°C, although occasionally two throat swabs were used to accommodate children who could not tolerate a nasal swab. These were batched and sent to the AFRIMS laboratory in Bangkok for diagnostic testing using influenza real-time reverse transcriptase polymerase chain reaction (rtRT-PCR) using primers and probes developed by the U.S. CDC [11].

A case test-negative study design was used post-hoc to evaluate VE in which vaccination coverage among those seeking care with ILI and testing positive by rtRT-PCR (cases) was compared with those testing negative (test-negative controls). The study was approved by the Institutional Review Boards of PMK and the Walter Reed Army Institute of Research. Written informed consent was obtained from adult subjects or the parents of child subjects. Informed assent was obtained from children ≥7 to <18 years old.

### Statistical Analysis

The study population was characterized by influenza PCR-positivity as well as influenza virus type and sub-type, and influenza vaccination status in the 12 months prior to illness. Vaccination status and vaccination date were based solely on the report of the subject. Those who reported being vaccinated within 14 days of fever onset were considered unvaccinated. The population was additionally characterized by gender, age category (6 to 23 months, 2 to 17, 18–49, 50–64, and 65 plus years of age), underlying disease, exposure to another person with similar symptoms, outpatient vs. inpatient status, and time from illness onset to respiratory sample collection. Age groupings were based on populations at risk for severe disease (children between 6 and 23 months and elderly adults age 65 and older) [12]. Other age groupings were intended to reflect school age (2 to17 years) and younger (18–49) vs. older adults (50–65). Associations between nominal categorical demographic characteristics and both influenza vaccination status and influenza PCR-positivity were evaluated using a chi-squared test. Associations between ordinal categorical characteristics and both influenza and vaccination status were evaluated with the Cochran-Mantel-Haenszel correlation statistic. Student’s t-test was used for variables that were at least approximately normally distributed. The Wilcoxon rank-sum test was used to evaluate days between onset of symptoms and specimen collection in relation to influenza and vaccination status.

Logistic regression models were employed to estimate the vaccination odds ratio (i.e., odds of vaccination in cases compared to test-negative controls) and estimated vaccine effectiveness using the formula VE = (1—vaccination odds ratio) * 100% [13]. Both unadjusted and adjusted models were used. Adjusted models included age (in years) using a recursive spline to account for the non-linearity of age in relation to incidence of influenza virus infection, as well as week of admission. In addition to calculating the overall VE for the entire study period, we also provide the VE estimate for individual May to April years after the epidemic that corresponds to the typical seasonal influenza experience where the southern hemisphere vaccine is employed. An estimate of the VE from the beginning of May 2010 through the end of the study period provides a summary estimate during a time when there was no vaccine strain change and excludes the time during which a mismatch existed between the vaccine and circulating H1N1 virus due to the pandemic. Additionally, we evaluated VE in sub-strata including age category. Very young children from 6 to 23 months of age were considered separately from older children because the very young children are a target group for vaccination according to the WHO and Thai government influenza vaccination recommendations. Persons ≥65 years old were considered separately from other adults for the same reason. Because VE has been observed to be lower in household-acquired vs. community-acquired influenza, we evaluated VE among those with and without exposure to another person with similar symptoms [14]. We also evaluated VE across strata of influenza virus type/subtype, presence of underlying disease, and outpatient vs. inpatient status. VE was evaluated across several high and low influenza activity periods defined a priori based on the percentage of influenza-like illness that was positive for influenza in published data from the Thailand National Influenza Center weekly influenza reports [15]. We identified high activity periods as having at least 25% of ILI being positive for influenza for at least 3 of 4 consecutive weeks. The activity periods correspond to the following periods: shortly after the start of the pandemic in Thailand (after the PMK surveillance began on Aug 23, 2009) through the end of 2009 (epiweek 2009–34 to 52); early 2010 through a high activity period ending in May 2010 (epiweek 2010–01 to 2010–21); another high activity period between May 30, 2010 and Oct 31, 2010 (epiweek 2010–22 through 2010–44); a low activity period between Nov 7, 2010 and 29 May, 2011 (epiweek 2010–45 to 2011–22); a high activity period between June 5, 2011 and Dec 25, 2011 (epiweek 2011–23 to 2011–52); a low activity period between Jan 1, 2012 and June 10, 2012 (epiweek 2012–1 to 2012–24); and a high activity period between June 17, 2012 and Jan 13, 2013 (epiweek 2012–25 to 2013–03). Finally, among those with sufficient information on the date of vaccination (to include at least both month and year), we calculated VE for vaccination occurring within 3, 6, 9 and 12 months prior to illness (relative to unvaccinated individuals). Trend analysis was evaluated using logistical regression with time since vaccination as a single ordinal variable. All statistical analyses were performed using R version 3.0.2 (R Foundation for Statistical Computing, Vienna, Austria).

## Results

**Fig 1:**
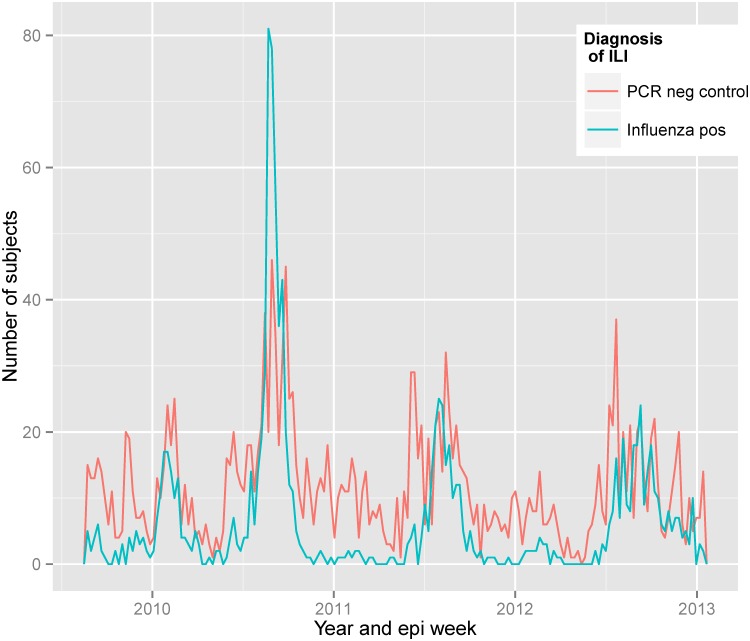
Distribution of influenza cases and test-negative controls from ILI patients at PMK hospital between late August 2010 and January of 2013.

Between August 25, 2009 and January 13, 2013, we enrolled and obtained specimens on 3,224 individuals with ILI. Of these, 1,133 tested positive by rRT-PCR for influenza (cases). The remaining 2,091 served as test-negative controls (Fig 1). Vaccination information was missing on 225 subjects: 74 cases (6.5% of all cases) and 151 controls (7.2% of test-negative controls) primarily from the 2 to 18 year age group. Compared to those with vaccine information, those without vaccine information were similarly likely to test positive for influenza (p = 0.46; p = 0.57 within the 2 to 18 age group). The remaining 2999 individuals available for analysis included 1059 (35.3%) cases and 1940 (64.7%) test-negative controls.

**Fig 2:**
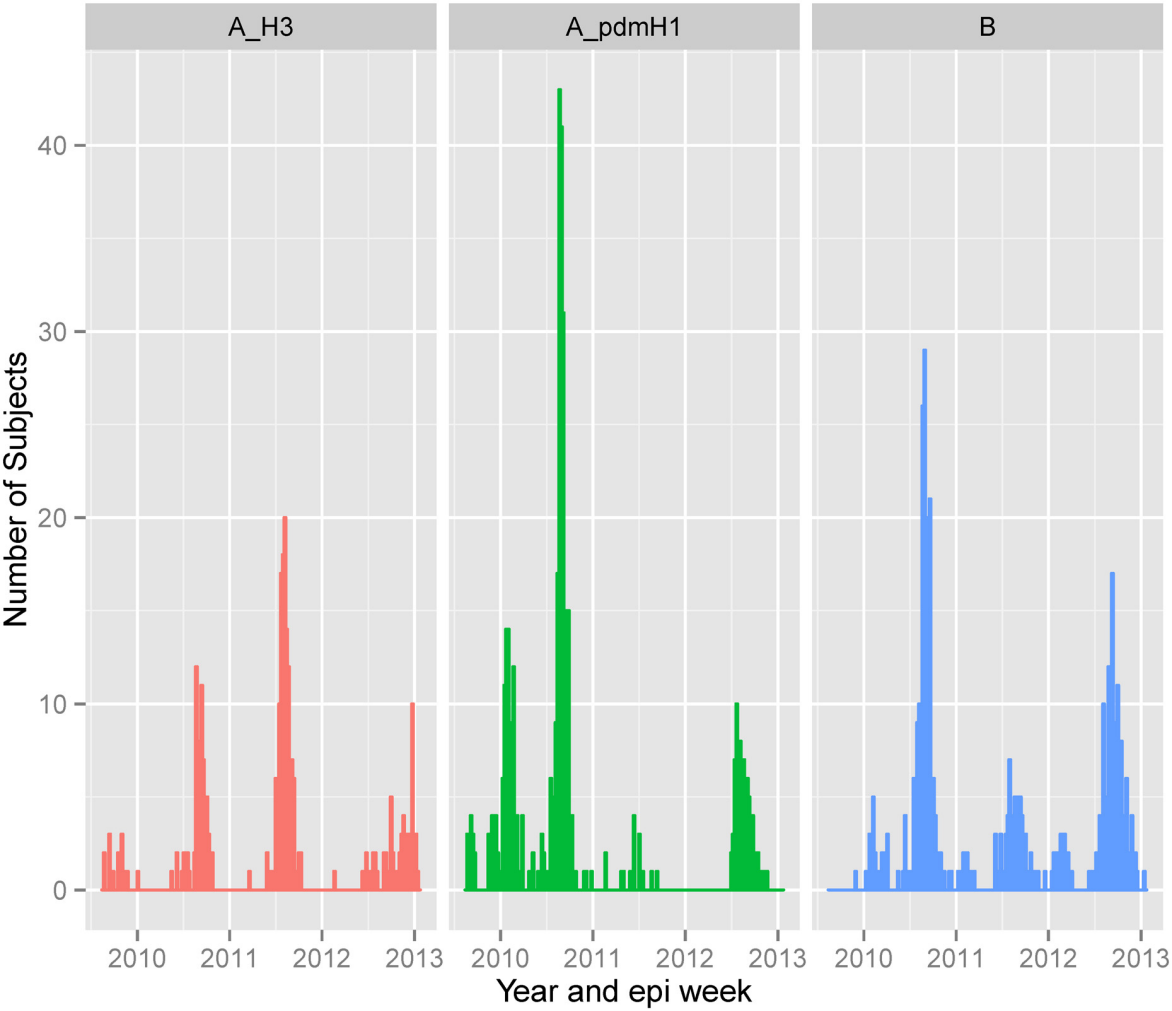
Distribution of influenza case type/subtype between late August 2010 and January of 2013.

The first influenza case in our surveillance occurred on August 25, 2009. Of the 1058 cases over the course of the surveillance, 421 (39.8%) had influenza A(H1N1)pdm09, 246 (23.2%) influenza A(H3N2) and 392 (37.0%) influenza B. Influenza A(H1N1)pdm09 was the predominant influenza A subtype during the early part of surveillance beginning in August 2009 through a period of high activity between January and May 2010 (Fig 2). Subsequently, a high influenza activity period between June and October corresponding to the rainy season included both influenza A(H1N1)pdm09 and A(H3N2) along with influenza B. Following this, a period of low influenza activity followed until June 2011. The subsequent increase in influenza activity which lasted until the end of the year was predominantly due to A(H3N2) and influenza B. A period of relatively little influenza activity predominated by influenza B occurred during the first half of 2012. During the peak influenza period of late June 2012 through the end of the year, influenza A(H1N1)pdm09, A(H3N2) as well as influenza B co-circulated. The dominant strains and seasonality of influenza was almost identical with that observed across other hospitals in Bangkok [16].

Influenza cases were slightly more likely than test-negative controls to be male. Influenza cases were also more likely to be in the adult age categories 18–49, 50–64 and ≥65 years of age (table 1). The 18–49 year old subjects were the most likely to be cases (52%). Test-negative controls were more likely than cases to be between 6 months and 23 months old; only 12.5% of these children were cases. Cases were more likely than test-negative controls to have been exposed to someone with similar symptoms and to be outpatients. Thirty-seven percent of outpatients were cases compared to only 17.2% of inpatients. Among outpatients, the test specimens were collected sooner after the onset of symptoms in cases (median 1.5 days) than test-negative controls (2.0 days) suggesting a small amount of misclassification from false negatives due to delayed testing (P = 0.007; data not shown). Cases did not differ from test-negative controls in their likelihood of having underlying disease (respiratory, heart, hematological, and renal).

**Table 1:** Univariate characteristics of PMK influenza surveillance subjects enrolled between August 2009 and January 2013.

	Influenza Cases		Test-Neg controls			Vaccinated		Unvaccinated		
Characteristics	N = 1059	35.3%	N = 1940	64.60%	P—value	N = 507	16.90%	N = 2492	83.00%	P—value
Gender					0.05*					0.11*
Female	456	43.1	906	46.8		247	48.7	1115	44.9	
Male	601	56.9	1029	53.2		260	51.3	1370	55.1	
Unknown	2		5			0		7		
Age group					<0.0001					<0.0001
6–23 months	73	6.9	513	26.4		96	18.9	490	19.7	
2 to 17 yrs	687	64.9	1136	58.6		358	70.6	1465	58.8	
18–49 yrs	256	24.2	237	12.3		37	7.3	456	18.3	
50 to 64 yrs	34	3.2	43	2.2		12	2.4	65	2.6	
65 plus yrs	9	0.8	11	0.6		4	0.8	16	0.6	
Underlying disease					0.98 *					<0.0001
Yes	170	16.1	312	16.1		139	27.5	343	13.8	
No	887	83.9	1624	83.9		367	72.5	2144	86.2	
Unknown/Missing	2		4			1		5		
Exposure to similar symptoms					<0.0001					0.93
Yes	503	47.7	743	38.4		209	41.6	1038	41.8	
No	551	52.3	1191	61.6		294	58.5	1448	58.2	
Inpt vs Outpt					<0.0001					0.41
OPD	1023	96.6	1767	91.1		476	93.9	2314	92.9	
IPD	36	3.4	173	8.9		31	6.1	178	7.1	
Time period					<0.0001					<0.0001
Aug 2009—Dec 2009	46	4.3	190	9.8		32	6.3	204	8.2	
Jan 2010—May 2010	114	10.8	168	8.7		34	6.7	248	10.0	
May 2010—Oct 2010	443	41.8	453	23.4		97	19.1	799	32.1	
Nov 2010—May 2011	22	2.1	255	13.1		67	13.2	210	8.4	
June 2011—Dec 2011	179	16.9	380	19.6		133	26.2	426	17.1	
Jan 2012—June 2012	21	2	124	6.4		37	7.3	108	4.3	
Jun 2012—Jan 2013	234	22.1	370	1901		107	21.3	497	19.8	

* P-value from Chi-square test does not include missing category

Of the 2999 with information on vaccination, 581 indicated they had been vaccinated in the last 12 months with varying levels of detail on the date of vaccination. Thirty-seven subjects (12 cases and 25 test-negative controls) were vaccinated within 14 days prior to illness and were, therefore, categorized as unvaccinated. Another 37 subjects (15 cases and 22 test-negative controls) reported vaccination dates more than 365 days prior to illness and were, therefore, categorized as unvaccinated (n = 37). For the current analysis, the remaining 507 were categorized as vaccinated. Of these, 417 (82.2%) subjects provided sufficient detail (at least month and year) that allowed characterization of immunization over quarterly intervals in the 12 months prior to illness.

Vaccinated subjects were more likely than unvaccinated subjects to be in the 2 to 17 year and ≥65 year age categories (table 1). Unvaccinated subjects were more likely to be 18 to 49 years old. Vaccinated subjects were more likely to have underlying disease. Vaccination status was similar with respect to gender, exposure to those with similar symptoms, outpatient status and the time between onset of symptoms and the collection of the test sample (P = 0.95; data not shown). The proportion vaccinated within the previous twelve months differed during different influenza activity periods. In the first three periods extending through October 2010, the proportion vaccinated was 13.6, 12.1, and 10.8%, respectively. Over the next three activity periods from November 2010 through June 2012, the proportion vaccinated was 24.3, 23.8 and 25.5%, respectively. During the final high influenza activity period between June 2012 and Jan 2013, the proportion was only 18.0%.

Antigenic characterization of influenza virus strains was not available for most study samples. Table 2 shows the circulating strains in Thailand (Thailand MOPH data) relative to the Southern Hemisphere trivalent inactivated vaccine strains during the years that would include the surveillance period. For anyone receiving vaccination before May 2010, the A(H1N1) component of the vaccine would likely have been a mismatch to the circulating A(H1N1)pdm09 strain. Afterward, however, the influenza A(H1N1) component of the vaccine would have been well matched to the circulating strain for the remainder of the surveillance. Circulating A(H3N2) in Thailand included some that would be a mismatch for the A(H3N2) vaccine component in 2009 and 2012. Circulating influenza B included strains and lineages not included in the vaccine during most of the study years.

**Table 2:** Circulating influenza virus strains relative to trivalent inactivated vaccine strain in Thailand including the study period.

	Circulating Strains			Trivalent Vaccine Formulation
Year (Thai Year)	A(H1N1)	A(H3N2)	B	Southern
2009 (2552)	A/California/07/2009 (82%)	A/Brisbane/10/2007 (68%)	B/Brisbane/60/2008 V (77%)	A/Brisbane/59/2007 (H1N1)
	A/Brisbane/59/2007 (18%)	A/Perth/16/2009 (32%)	B/Malaysia/2506/2004 V (32%)	A/Brisbane/10/2007 (H3N2)
				B/Florida/4/2006 V
2010 (2553)	A/California/07/2009(100%)	A/Perth/16/2009 (100%)	B/Brisbane/60/2008 V (89%)	A/California/7/2009 (H1N1)
			B/Malaysia/2506/2004 V (11%)	A/Perth/16/2009(H3N2)
				B/Brisbane/60/2008 V
2011 (2554)	A/California/07/2009(100%)	A/Perth/16/2009 (100%)	B/Brisbane/60/2008 V (93%)	A/California/7/2009 (H1N1)
			B/Florida/60/2008 V (6%)	A/Perth/16/2009(H3N2)
			B/Wisconsin/01/2010 Y (1%)	B/Brisbane/60/2008 V
2012 (2555)	A/California/07/2009(100%)	A/Perth/16/2009 (58%)	B/Brisbane/60/2008 V (68%)	A/California/7/2009 (H1N1)
		A/Victoria/361/2011 (42%)	B/Florida/60/2008 V (1%)	A/Perth/16/2009(H3N2)
			B/Wisconsin/01/2010 Y (31%)	B/Brisbane/60/2008 V

V = Influenza B Victoria linage; Y = Influenza B Yamagata lineage. Table adapted from Influenza viruses in Thailand: 7 years of sentinel surveillance data, 2004–2010, Chittaganpitch et al. Influenza and Other Respiratory Viruses DOI:10.1111/j.1750-2659.2011.00302.x.(years 2009–2010) and updated with data from http://www.thainihnic.org/influenza/main.php?option=newsletter (years 2011 to 2013). Note: Northern Hemisphere vaccine strains did not differ from southern vaccine strains after the inclusion of A/California/7/2009(H1N1) in April 2010.

### Influenza Vaccine Effectiveness

The overall adjusted vaccine effectiveness was estimated to be 50.1% (95% CI: 35.0 to 61.9%)(table 3.). The May to April VE for year 2010, 2011 and 2012 was 57.7% (95%CI: 33.7, 73.8%), 57.1% (95% CI: 35.2, 68.3%) and 37.6% (95% CI: 3.5, 62.9%), respectively (S1 Table. Influenza VE May 2010 to April 2011; S2 Table. Influenza VE May 2011 to April 2012; S3 Table. Influenza VE May 2012 to January 2013). The VE for the entire post-pandemic period beginning May 2010 to the end of the surveillance during which the vaccine would have been a good match to the circulating influenza A (H1NI) was 52.6% (95% CI: 38.0, 64.0%) (S4 Table. Influenza VE May 2010 to January 2013).

**Table 3:** Estimates of Influenza vaccine effectiveness for subject in PMK surveillance between August 2009 and January 2013.

	Influenza Positive		Influenza Negative		Vaccine Effectiveness			
	No. vacc	Pct vacc	No. vacc	Pct vacc	Unadjusted	95% CI	Adjusted *	95% CI
All	113 / 1059	10.7	394/ 1940	20.3	53.1	41.5,62.7	50.1	35.0,61.9
Age group								
6–23 months	7 / 73	9.6	89 / 513	17.3	49.5	-6.8,79.5	58.1	-5.7,85.2
2 to 17 yrs	91 / 687	13.2	267/1136	23.5	50.3	35.8, 61.8	52.5	35.6, 65.1
18–49 yrs	11/256	4.3	26 / 237	11.0	63.6	26.3,83.1	76.6	40.1, 91.6
50 to 64 yrs	2 / 34	5.9	10 / 43	23.3	79.4	14.1,97.0	**	
65 plus yrs	2 / 9	22	2/11	18.2	-28.6	-1207,87.3	**	
Influenza virus type/subtype								
A(H1N1)pdm09	31/ 421	7.4	394/1940	20.3	68.8	55.0, 79.1	59.8	38.3,74.6
A(H3N2)	36/ 246	14.6	394 /1940	20.3	32.7	3.8,54.2	46.5	17.6, 66.0
B	46 / 391	11.8	394 /1940	20.3	47.8	28.4, 62.8	44.1	17.5, 62.7
Underlying Disease								
Yes	30 / 170	17.6	109/312	34.9	60.1	37.6,75.1	75.5	45.3,89.5
No	82/887	9.2	285/1624	17.5	52.1	38.2,63.3	48.0	29.7, 61.8
Exposure to similar symptoms								
Yes	55 / 504	10.9	154 / 743	20.7	53.1	35.1,66.6	55.6	32.2,71.3
No	56 / 551	10.2	238 /1191	20.0	54.7	38.6, 67.1	49.2	25.7,65.7
Inpatient vs Outpatient								
OPD	110 / 1023	10.8	366 / 1767	20.7	53.8	42.2, 63.5	49.1	33.2, 61.3
IPD	3 / 36	8.3	28 / 173	16.2	52.9	-43.4,89.2	‡	
Time period								
Aug 2009—Dec 2009	5 / 46	10.9	27 / 190	14.2	26.4	-88.5,76.2	20.6	-143.0,77.5
Jan 2010—May 2010	10 / 114	8.8	24 / 168	14.3	42.3	-22.6,74.6	47.5	-20.1, 78.4
May 2010—Oct 2010	31 / 443	7.0	66 / 453	14.6	55.8	31.5, 72.2	55.1	25.4,73.4
Nov 2010 to May 2011	4 / 22	18.2	63 / 255	24.7	32.3	-89.6,81.0	32.0	-132.0, 83.8
June 2011—Dec 2011	26/179	14.5	107/380	28.2	56.6	31.4,73.4	59.7	30.8, 77.1
Jan 2012—June 2012	6 /21	28.6	31 / 124	25.0	-20.0	-224, 60.1	-48.1	-430.0, 60.3
Jun 2012—Jan 2013	31/234	13.2	76/ 370	20.5	40.9	7.8, 62.9	47.0	9.8, 69.4

* Adjusted for age using recursive spline and epiweek

** Model produced infinite or undefined confidence intervals

^‡^ Model did not converge

Among the different age strata, VE was highest in the 18–49 year age group (VE = 76.6%; 95% CI: 40.1 to 91.6%). Adjusted estimates were not done for adults older than 49 years due to small numbers relative to the variables in the model which included epiweek. However, the unadjusted estimates suggest that VE was high among the 50 to 64 year group, but very low in those ≥65 years old. The adjusted VE point estimate was slightly lower in the 2–17 year age group (52.5%) relative to the 18–49 year age group and the 6–23 month age category (58.1%). The vaccine recommendation for very young children less than 2 years of age has sometimes been interpreted to include 2 year olds. For this reason, we also evaluated VE using an alternative definition for the youngest age category to include those between 6 and 35 months of age. The adjusted VE was 56.0% (95%CI: 14.8, 78.7) in this age range. Additional adjustment to the overall VE estimate for gender, underlying disease, time between symptom onset and specimen collection, and outpatient vs inpatient status, did not change the results (VE: 50.0% 50.9%, 50.3% and 50.5%, respectively) and were left out of the model.

VE estimates did not vary remarkably for the specific influenza virus type/sub-types. The highest point estimate of VE was that for infection with A(H1N1)pdm09 (59.8%). After the pandemic (beginning May 2010) however, it was 68.7% (95% CI: 46.1, 82.7%). VE was similar for seasonal influenza A(H3N2) (46.5%) and influenza B (44.1%). However, the VE for A(H3N2) and B appeared to vary over the individual years. During the May/April period beginning in 2010, the A(H3N2) VE was -0.7 (95% CI: -118, 57.6). During the May/April period beginning 2012, the VE for influenza B was -2.7 (95%CI: -101, 48.4).

VE appeared to be higher for those with underlying disease (75.5%; 95% CI:45.3 to 89.5%) compared to those without underlying disease (48.0%; 95%CI:29.7 to 61.8%). VE appeared similar between those that were (55.6%) and were not (49.2%) exposed to someone with similar symptoms. Unadjusted VE appeared similar between outpatients and inpatients. However, small numbers prevented the estimate of a reliable adjusted VE in the inpatient group.

We evaluated VE across different influenza activity periods during the surveillance period. Although the estimates were particularly unstable during low activity periods, VE estimates appeared much higher during high versus low influenza activity periods. VE during low activity periods from August to December 2009, November 2010 to May 2011, and January to June 2012 was 20.6, 32.0 and -48.1%, respectively. These were considerably lower than the point estimates for high influenza activity periods that occurred during the typical (for Thailand) peak influenza seasons corresponding to just after the onset of the rainy season in 2010, 2011, and 2012. VE during these periods was estimated to be 55.1, 59.7 and 47.0%, respectively. We aggregated the above periods into three categories: the global second wave of the pandemic (Aug 2009 to May 2010); low influenza activity periods (Nov 2010 to May 2011 and Jan to June 2012); and the remaining periods that constituted high influenza activity periods (May to Oct 2010, June to Dec 2011, and June 2012 to Jan 2013). The VE estimates for these aggregated periods were 39.0% (95%CI: -19.2, 70.1), 4.4% (95% CI: -126.5, 62.2) and 54.9% (95% CI: 38.9, 66.9), respectively.

We also evaluated the relationship between VE and time between vaccination and illness. For this analysis, we excluded 90 individuals reporting vaccination within the previous 12 months, but not reporting vaccine date with sufficient detail to measure the interval (i.e., did not report at least the month and year of vaccination). We also excluded the 74 subjects classified as not vaccinated who reported vaccination within 14 days or more than 365 days of the onset of illness. VE was highest among those reporting vaccination between 14 days and 3 months prior to illness (58.5%; 95%CI 30.8, 75.9). VE tended to decrease with progressively longer intervals between vaccination and illness with VE estimates of 47.2% (95% CI: 11.3, 69.3), 44.6% (9%% CI:6.5, 68.0) and 25.5 (95%CI: -40.8, 61.6) for intervals of >3 to 6, >6 to 9 and >9 to 12 months, respectively (P for trend = 0.00001 includes the unvaccinated as highest in ordinal category of time since vaccination after >9 to 12 months; data not shown). However, evaluating the trend only among those reporting vaccination within the last year (with the > 9–12 month category as the referent), the P for trend was only 0.44. Excluding the pandemic period, the p for this trend was 0.56.

## Discussion

Self-reported vaccination within 12 months prior to illness was associated with a 50.1% (95% CI: 35.0 to 61.9%) reduced risk of medically attended influenza in this population of persons seeking care at a military hospital in urban Bangkok. Yearly estimates corresponding to evaluation of the southern hemisphere seasonal influenza vaccine for the years 2010, 2011 and 2012 were 57.7(95%CI: 33.7, 73.8%), 57.1% (95% CI: 35.2, 68.3%) and 37.6% (95% CI: 3.5, 62.9%),

Few reports from tropical regions are available for comparison. However, our finding of 58.1% VE in 6 to 23 month old children is similar to those estimated for fully vaccinated children in a cohort of healthy and high risk children at a large public hospital in Bangkok over a similar time frame (56% in 2011–2012 and 64% in 2012–2013) [17]. Another estimate of inactivated influenza vaccine effectiveness against hospitalization among adults age 50 years and older in rural Thailand during June to December of 2010 and 2011 was 47% (95% CI: 5–71%) [18]. Among groups at risk for severe complications from influenza, we found support for the utility of vaccination among children <2 years old as well as persons with underlying disease. Small sample sizes made it difficult to evaluate those ≥65 years old.

Overall, our results suggest that vaccination provides moderate protection against influenza in the tropics. Comparison with results of VE studies of inactivated vaccine against medically attended, laboratory confirmed influenza from temperate regions are difficult for the reason that these studies are widely variable due to differences in study size, study population, incidence of influenza over place and season, and the match between circulating and vaccine strains [19]. However, such studies are also largely consistent with moderate protection with VE estimates between 56 and 62% [19–25].

Estimates of VE were low during the first few months of surveillance that corresponded to the beginning of the second wave of the 2009 influenza pandemic (between September 2009 and Dec 2009). During this period, influenza vaccine that matched the A(H1N1)pdm09 strain that predominated during this period was largely unavailable in Thailand. In Europe and Canada, the median VE estimate for the monovalent pandemic vaccine was 69% [19]. However, in our study, those with influenza during this period who reported being vaccinated during the prior 12 months would largely have received the previous seasonal trivalent inactivated vaccine lacking the pandemic strain. Beginning in May 2010, however, the seasonal trivalent vaccine would have included A(H1N1)pdm09 for the remainder of the surveillance period. This may explain why, in spite of the poor VE at the beginning of surveillance during the early phase of the pandemic, the overall VE was higher for A(H1N1)pdm09 (59.8%) than A(H3N2) (46.5%) and B (41.1%). In the post pandemic period, the VE for A(H1N1)pdm09 infection was 68.7%. Unlike A(H1N1), A(H3N2) and B had other strains circulating that were not included in the vaccine during the surveillance period, possibly accounting for diminished VE due to antigenic mismatch. Such an antigenic mismatch may have occurred with influenza A(H3N2) and B infections in 2010 and 2012,respectively.

Notably, VE was low during the low influenza activity periods. This may be due to a poor match between the vaccine and the circulating viruses during these low periods. Perhaps low influenza activity allows for more diverse sporadic virus populations such as those that occasionally seed larger subsequent outbreaks. Alternatively, low VE estimates during these low activity periods may arise from the misclassification of influenza illnesses. The case test-negative study design can be subject to bias due to imperfect sensitivity and specificity of influenza tests. Our use of highly specific rRT-PCR assays for virus detection would have minimized this potential bias. However, the bias may not be trivial during low-influenza incidence periods. Relative to high incidence periods, imperfect sensitivity during low incidence periods can enrich the unvaccinated population with more false-negatives relative to the vaccinated population leading to an underestimation of VE [26].

The case test-negative design employed in this analysis has an advantage over other observational study designs in reducing bias arising from differences in health care seeking behavior by taking both cases and controls from among those seeking care for ILI. Nonetheless, a limitation to all case test-negative studies remains. If disease severity differed between influenza and non-influenza illnesses, and if severity was related to the subject seeking health care and, in turn, health care seeking was related to vaccination, then we can expect VE to be overestimated when influenza is more severe than the non-influenza illnesses [13]. Such bias requires adjustment for severity. In our study, small numbers of inpatients and poor characterization of severity of disease prevents us from adjusting for severity.

Our reliance on self-reporting for vaccination data is a limitation of this study given the potential for recall bias. The data did not permit us to evaluate the VE of full vs. partial vaccination. We were also unable to evaluate whether VE was higher in those with no influenza vaccinations prior to the 12 month period before illness relative to those with multiple previous vaccinations as has been observed in at least one study [14],. However, the influenza vaccination program in Thailand was only recently scaled up to include a meaningful number of persons at risk for complications from influenza. It is, therefore, likely that many of our subjects were vaccinated for the first time. It is also possible that those with underlying disease may have been more likely to have been vaccinated before and such priming may be the reason for the higher VE seen relative to healthier patients. In contrast, some investigators have found reduced VE with repeated seasonal influenza vaccination. These findings, however, would require further investigation.

Interestingly, we found that VE became lower as the time interval between vaccination and illness became longer during the 12 month period prior to illness. A VE estimate of 58.5% when the time interval was ≤3 months decreased to 25.5% when the interval was >9 to 12 months. This suggests a reduction in either strain-specific immunity induced by matched inactivated vaccine or cross-protective immunity by slightly mismatched vaccine over the course of a year. If corroborated in future studies, there may be a role for booster vaccinations before 12 months even when the vaccine formulation does not change. Alternatively, the decrease in VE may be due to mismatches between vaccine and infecting strains over time.

Unlike the temperate climates, our data confirm that influenza in Thailand, like other tropical countries, persists at variable levels throughout the year. Sentinel surveillance data from Thailand show that more than one circulating strain exists in many years. We could not directly evaluate whether circulating strains were more variable in this region with year round virus circulation compared to temperate regions, and what effect this would have on vaccine effectiveness relative to temperate climates. Further studies with comprehensive antigenic characterization of circulating virus strains and more detailed vaccination characterization would be beneficial. Our results demonstrate moderate protection by influenza vaccination, and support the value of vaccination in the tropics to prevent a significant cause of morbidity and mortality particularly in very young children and those with underlying disease. As vaccination programs continue to expand in tropical regions, selection pressures from changes in population level immunity may have an impact on the global ecology of influenza viruses. This underscores the value of influenza surveillance and vaccine efficacy studies in Southeast Asia.

## Supporting Information

S1 Table Influenza VE May 2010 to April 2011. (DOCX)

S2 Table Influenza VE May 2011 to April 2012. (DOCX)

S3 Table Influenza VE May 2012 to January 2013. (DOCX)

S4 Table Influenza VE May 2010 to January 2013. (DOCX)

S5 Table Influenza VE August 2009 to January 2013 among outpatients only. (DOCX)
