# Supplementary material for: Influenza Vaccine Effectiveness in the Tropics: Moderate Protection in a Case Test-Negative Analysis of a Hospital-Based Surveillance Population in Bangkok between August 2009 and January 2013
Source: PLoS One. 2015 Aug 12;10(8):e0134318. doi: 10.1371/journal.pone.0134318 (PMC4534293; doi:10.1371/journal.pone.0134318)
Supplement: S1 Table — (DOCX) [file pone.0134318.s001.docx]

|  | Influenza Positive | |  | Influenza Negative | |  | Vaccine Effectiveness | | | | |
| --- | --- | --- | --- | --- | --- | --- | --- | --- | --- | --- | --- |
|  | No. vacc | Pct vacc |  | No. vacc | Pct vacc |  | Unadjusted | 95% CI |  | Adjusted * | 95% CI |
| All | 33/465 | 7.1 |  | 120/690 | 17.4 |  | 63.7 | 46.2,76.1 |  | 57.7 | 33.7,73.8 |
| Age group |  |  |  |  |  |  |  |  |  |  |  |
| 6-23 months | 1/26 | 3.8 |  | 30/194 | 15.5 |  | 78.1 | -9.5,98.8 |  | 75.6 | -74.9,98.8 |
| 2 to 17 yrs | 26/290 | 9 |  | 76/397 | 19.1 |  | 58.4 | 34.0,74.5 |  | 56.7 | 24.6,75.6 |
| 18-49 yrs | 6/132 | 4.5 |  | 11/85 | 12.9 |  | 68 | 12.2,89.3 |  | 61.2 | -30.1,89.0 |
| 50 to 64 yrs | 0/13 | 0 |  | 2/10 | 20.0 |  | ** |  |  | ** |  |
| 65 plus yrs | 0/4 | 0 |  | 1/4 | 25.0 |  | ** |  |  | ** |  |
|  | |  |  |  |  |  |  |  |  |  |  |
| Influenza virus type/subtype | |  |  |  |  |  |  |  |  |  |  |
| A(H1N1)pdm09 | 11/217 | 5.1 |  | 120/690 | 17.4 |  | 74.6 | 54.1,87.3 |  | 71.1 | 41.8,86.7 |
| A(H3N2) | 9/69 | 13 |  | 120/690 | 17.4 |  | 28.8 | -40.6,67.8 |  | -0.7 | -118.6,57.5 |
| B | 13/179 | 7.3 |  | 120/690 | 17.4 |  | 62.8 | 34.7,80.4 |  | 53 | 8.9,77.2 |
|  |  |  |  |  |  |  |  |  |  |  |  |
| Underlying Disease |  |  |  |  |  |  |  |  |  |  |  |
| Yes | 11/79 | 13.9 |  | 30/116 | 25.9 |  | 53.6 | 3.2,79.1 |  | 63.4 | -42.0,91.0 |
| No | 22/386 | 5.7 |  | 90/573 | 15.7 |  | 67.6 | 48.2,80.4 |  | 61.7 | 33.3,78.7 |
|  | |  |  |  |  |  |  |  |  |  |  |
| Exposure to similar symptoms | |  |  |  |  |  |  |  |  |  |  |
| Yes | 9/206 | 4.4 |  | 45/255 | 17.6 |  | 78.7 | 57.2,90.5 |  | 69.6 | 24.5,88.6 |
| No | 24/259 | 9.3 |  | 75/435 | 17.2 |  | 51 | 21.2,70.5 |  | 50.3 | 9.5,73.4 |
|  |  |  |  |  |  |  |  |  |  |  |  |
| Inpatient vs Outpatient |  |  |  |  |  |  |  |  |  |  |  |
| OPD | 32/438 | 7.3 |  | 96/571 | 16.8 |  | 61 | 41.2,74.8 |  | 48.6 | 14.7,69.5 |
| IPD | 1/27 | 3.7 |  | 24/119 | 20.2 |  | 84.8 | 22.4,99.2 |  | 95.2 | 60.5,99.8 |

* Adjusted for age using recursive spline and epiweek

** Problems with convergence, failure to converge or perfect separation.
