# Supplementary material for: Influenza Vaccine Effectiveness in the Tropics: Moderate Protection in a Case Test-Negative Analysis of a Hospital-Based Surveillance Population in Bangkok between August 2009 and January 2013
Source: PLoS One. 2015 Aug 12;10(8):e0134318. doi: 10.1371/journal.pone.0134318 (PMC4534293; doi:10.1371/journal.pone.0134318)
Supplement: S2 Table — (DOCX) [file pone.0134318.s002.docx]

|  | Influenza Positive | |  | Influenza Negative | |  | Vaccine Effectiveness | | | | |
| --- | --- | --- | --- | --- | --- | --- | --- | --- | --- | --- | --- |
|  | No. vacc | Pct vacc |  | No. vacc | Pct vacc |  | Unadjusted | 95% CI |  | Adjusted * | 95% CI |
| All | 32/202 | 15.8 |  | 142/511 | 27.8 |  | 51.1 | 26.1,68.4 |  | 57.0 | 35.2,68.3 |
| Age group |  |  |  |  |  |  |  |  |  |  |  |
| 6-23 months | 2/25 | 8 |  | 30/134 | 22.4 |  | 69.9 | -10.1,95.3 |  | 82.9 | 17.3,98.0 |
| 2 to 17 yrs | 25/137 | 18.2 |  | 107/351 | 30.5 |  | 49.1 | 18.0,66.3 |  | 58.9 | ‡ |
| 18-49 yrs | 4/31 | 12.9 |  | 2/20 | 10 |  | -33.3 | -934,76.6 |  | ** |  |
| 50 to 64 yrs | 0/6 | 0 |  | 3/5 | 60 |  | ** |  |  | ** |  |
| 65 plus yrs | 1/3 | 33.3 |  | 0/1 | 0 |  | ** |  |  | ** |  |
|  | |  |  |  |  |  |  |  |  |  |  |
| Influenza virus type/subtype | |  |  |  |  |  |  |  |  |  |  |
| A(H1N1)pdm09 | 2/12 | 16.7 |  | 142/511 | 27.8 |  | 48 | -100,92.1 |  | ** |  |
| A(H3N2) | 18/114 | 15.8 |  | 142/511 | 27.8 |  | 51.3 | 18.3,72.4 |  | 59.1 | 33.7,70.0 |
| B | 12/76 | 15.8 |  | 142/511 | 27.8 |  | 51.3 | 10.1,75.6 |  | 54.2 |  |
|  |  |  |  |  |  |  |  |  |  |  |  |
| Underlying Disease |  |  |  |  |  |  |  |  |  |  |  |
| Yes | 10/32 | 31.2 |  | 49/96 | 51 |  | 56.4 | 0.3,82.0 |  | 76.0 | 7.3,94.6 |
| No | 22/170 | 12.9 |  | 93/415 | 22.4 |  | 48.5 | 16.2,69.5 |  | 48.6 | 54.9,69.4 |
|  | |  |  |  |  |  |  |  |  |  |  |
| Exposure to similar symptoms | |  |  |  |  |  |  |  |  |  |  |
| Yes | 17/102 | 16.7 |  | 48/194 | 24.7 |  | 39.2 | -10.7,67.8 |  | 42.4 | -23.7,74.3 |
| No | 14/99 | 14.1 |  | 92/315 | 29.2 |  | 60.1 | 28.1,79.2 |  | ** |  |
|  |  |  |  |  |  |  |  |  |  |  |  |
| Inpatient vs Outpatient |  |  |  |  |  |  |  |  |  |  |  |
| OPD | 31/198 | 15.7 |  | 142/497 | 28.6 |  | 53.6 | 29.5,70.2 |  | 55.4 | ** |
| IPD | 1/4 | 25 |  | 0/14 | 0 |  | ** |  |  | ** |  |

* Adjusted for age using recursive spline and epiweek

** Problems with convergence, failure to converge or perfect separation.
