# Supplementary material for: Influenza Vaccine Effectiveness in the Tropics: Moderate Protection in a Case Test-Negative Analysis of a Hospital-Based Surveillance Population in Bangkok between August 2009 and January 2013
Source: PLoS One. 2015 Aug 12;10(8):e0134318. doi: 10.1371/journal.pone.0134318 (PMC4534293; doi:10.1371/journal.pone.0134318)
Supplement: S3 Table — (DOCX) [file pone.0134318.s003.docx]

|  | Influenza Positive | |  | Influenza Negative | |  | Vaccine Effectiveness | | | | |
| --- | --- | --- | --- | --- | --- | --- | --- | --- | --- | --- | --- |
|  | No. vacc | Pct vacc |  | No. vacc | Pct vacc |  | Unadjusted | 95% CI |  | Adjusted * | 95% CI |
| All | 33/236 | 14 |  | 81/392 | 20.7 |  | 37.6 | 3.7,60.3 |  | 39.6 | 3.5,62.9 |
| Age group |  |  |  |  |  |  |  |  |  |  |  |
| 6-23 months | 3/15 | 20 |  | 22/122 | 18 |  | -13.6 | -295,75.6 |  | -22.0 | ‡ |
| 2 to 17 yrs | 27/162 | 16.7 |  | 54/197 | 27.4 |  | 47 | 11.8,68.8 |  | 49.7 | 9.2,72.8 |
| 18-49 yrs | 1/51 | 2 |  | 4/60 | 6.7 |  | 72 | -97.0,98.6 |  | 91.2 | 76.0,99.5 |
| 50 to 64 yrs | 2/8 | 25 |  | 1/10 | 10 |  | ** |  |  | ** |  |
| 65 plus yrs | 0/0 |  |  | 0/3 |  |  | ** |  |  | ** |  |
|  | |  |  |  |  |  |  |  |  |  |  |
| Influenza virus type/subtype | |  |  |  |  |  |  |  |  |  |  |
| A(H1N1)pdm09 | 5/73 | 6.8 |  | 81/392 | 20.7 |  | 71.8 | 34.1,90.3 |  | 70.4 | 23.3,90.8 |
| A(H3N2) | 7/49 | 14.3 |  | 81/392 | 20.7 |  | 36.4 | -39.4,74.5 |  | 53.9 | -25.3,85.5 |
| B | 21/114 | 18.4 |  | 81/392 | 20.7 |  | 13.3 | -45.5,50.1 |  | -2.7 | -101.3,48.4 |
|  |  |  |  |  |  |  |  |  |  |  |  |
| Underlying Disease |  |  |  |  |  |  |  |  |  |  |  |
| Yes | 7/35 | 20 |  | 15/50 | 30 |  | 41.7 | -51.6,80.1 |  | 87.4 | 5.5,99.2 |
| No | 25/199 | 12.6 |  | 66/339 | 19.5 |  | 40.6 | 3.3,64.4 |  | 37.7 | -11.0,65.8 |
|  | |  |  |  |  |  |  |  |  |  |  |
| Exposure to similar symptoms | |  |  |  |  |  |  |  |  |  |  |
| Yes | 23/130 | 17.7 |  | 41/163 | 25.2 |  | 36 | -12.6,64.4 |  | 40.8 | 19.4,71.1 |
| No | 9/104 | 8.7 |  | 40/226 | 17.7 |  | 55.9 | 9.2,80.6 |  | 53.4 | -19.1,83.2 |
|  |  |  |  |  |  |  |  |  |  |  |  |
| Inpatient vs Outpatient |  |  |  |  |  |  |  |  |  |  |  |
| OPD | 32/234 | 13.7 |  | 81/386 | 21 |  | 40.3 | 7.6,62.3 |  | 43.1 | 3.8,66.92 |
| IPD | 1/2 | 50 |  | 0/6 | 0 |  | ** |  |  | ** |  |

* Adjusted for age using recursive spline and epiweek

** Problems with convergence, failure to converge or perfect separation.
