# Supplementary material for: Influenza Vaccine Effectiveness in the Tropics: Moderate Protection in a Case Test-Negative Analysis of a Hospital-Based Surveillance Population in Bangkok between August 2009 and January 2013
Source: PLoS One. 2015 Aug 12;10(8):e0134318. doi: 10.1371/journal.pone.0134318 (PMC4534293; doi:10.1371/journal.pone.0134318)
Supplement: S4 Table — (DOCX) [file pone.0134318.s004.docx]

|  | Influenza Positive | |  | Influenza Negative | |  | Vaccine Effectiveness | | | | |
| --- | --- | --- | --- | --- | --- | --- | --- | --- | --- | --- | --- |
|  | No. vacc | Pct vacc |  | No. vacc | Pct vacc |  | Unadjusted | 95% CI |  | Adjusted * | 95% CI |
| All | 98 /903 | 10.9 |  | 343/1593 | 21.5 |  | 55.6 | 43.7, 65.3 |  | 52.6 | 38.0,64.0 |
| Age group |  |  |  |  |  |  |  |  |  |  |  |
| 6-23 months | 6 / 66 | 9.1 |  | '82 / 450 | 18.2 |  | 55.1 | 0.4 , 83.1 |  | 63.8 | 3.8, 88.2 |
| 2 to 17 yrs | 78 / 589 | 13.2 |  | 237 / 945 | 25.1 |  | 54.4 | 39.9,65.7 |  | 54.0 | 36.1, 67.2 |
| 18-49 yrs | 11 / 214 | 5.1 |  | 17 / 165 | 10.3 |  | 52.8 | -2.5,79.1 |  | 65.9 | 3.9 , 88.6 |
| 50 to 64 yrs | 2 /27 | 7.4 |  | 6 / 25 | 24 |  | 74.7 | -24.0, 96.6 |  | ** |  |
| 65 plus yrs | 1 / 7 | 14.3 |  | 1 / 8 | 12.5 |  | -16.7 | -3303,96.0 |  | ** |  |
|  | |  |  |  |  |  |  |  |  |  |  |
| Influenza virus type/subtype | |  |  |  |  |  |  |  |  |  |  |
| A(H1N1)pdm09 | 18 / 302 | 6 |  | 343 / 1593 | 21.5 |  | 76.9 | 63.3,86.3 |  | 68.7 | 46.1,82.7 |
| A(H3N2) | 34 / 232 | 14.7 |  | 343 / 1593 | 21.5 |  | 37.4 | 9.4,58.0 |  | 46.6 | 16.7, 66.5 |
| B | 46 / 369 | 12.5 |  | 343 / 1593 | 21.5 |  | 48.1 | 28.4, 63.1 |  | 39.7 | 10.6,59.9 |
|  |  |  |  |  |  |  |  |  |  |  |  |
| Underlying Disease |  |  |  |  |  |  |  |  |  |  |  |
| Yes | 28 / 146 | 19.2 |  | 94 / 262 | 35.9 |  | 57.6 | 32.0, 74.2 |  | 73.7 | 37.4, 89.5 |
| No | 69 / 755 | 9.1 |  | 249 /1327 | 18.8 |  | 56.4 | 42.5, 67.4 |  | 51.0 | 31.9,65.0 |
|  | |  |  |  |  |  |  |  |  |  |  |
| Exposure to similar symptoms | |  |  |  |  |  |  |  |  |  |  |
| Yes | 49 / 438 | 11.2 |  | 134 / 612 | 21.9 |  | 55.1 | 36.4, 68.7 |  | 54.1 | 28.0, 71.1 |
| No | 47 / 462 | 10.2 |  | 207 / 976 | 21.2 |  | 57.9 | 41.5,70.3 |  | 52.6 | 28.3, 69.1 |
|  |  |  |  |  |  |  |  |  |  |  |  |
| Inpatient vs Outpatient |  |  |  |  |  |  |  |  |  |  |  |
| OPD | 95 / 870 | 10.9 |  | 319 / 1454 | 21.9 |  | 56.4 | 44.4, 66.1 |  | 50.3 | 33.4, 63.2 |
| IPD | 3 / 33 | 9.1 |  | 24 / 139 | 17.3 |  | 52.1 | -49.2, 89.1 |  | 92.3 | 68.5, 93.4 |

* Adjusted for age using recursive spline and epiweek

** Problems with convergence, failure to converge or perfect separation.
