# Supplementary material for: Influenza Vaccine Effectiveness in the Tropics: Moderate Protection in a Case Test-Negative Analysis of a Hospital-Based Surveillance Population in Bangkok between August 2009 and January 2013
Source: PLoS One. 2015 Aug 12;10(8):e0134318. doi: 10.1371/journal.pone.0134318 (PMC4534293; doi:10.1371/journal.pone.0134318)
Supplement: S5 Table — (DOCX) [file pone.0134318.s005.docx]

|  | Influenza Positive | |  | Influenza Negative | |  | Vaccine Effectiveness | | | | |
| --- | --- | --- | --- | --- | --- | --- | --- | --- | --- | --- | --- |
|  | No. vacc | Pct vacc |  | No. vacc | Pct vacc |  | Unadjusted | 95% CI |  | Adjusted * | 95% CI |
| All | 110 / 1023 | 10.8 |  | 366/ 1767 | 20.7 |  | 53.9 | 42.2,62.5 |  | 49.1 | 33.2,61.3 |
| Age group |  |  |  |  |  |  |  |  |  |  |  |
| 6-23 months | 7 / 65 | 10.8 |  | 85 / 447 | 19.0 |  | 48.6 | -9.5,79.2 |  | 55.0† | -16.7,84.4 |
| 2 to 17 yrs | 89 / 660 | 13.5 |  | 244/1040 | 23.5 |  | 49.2 | 33.9, 61.2 |  | 38.1† | 14.3, 55.5 |
| 18-49 yrs | 11/256 | 4.3 |  | 25 / 231 | 10.8 |  | 63.0 | 24.8,82.9 |  | 76.8† | 39.6, 91.8 |
| 50 to 64 yrs | 2 / 34 | 5.9 |  | 10 / 42 | 23.8 |  | 80.0 | 16.6,97.1 |  | ** |  |
| 65 plus yrs | 1 / 8 | 12.5 |  | 2/7 | 28.6 |  | 64.3 | -377,98.6 |  | ** |  |
| Influenza virus type/subtype | |  |  |  |  |  |  |  |  |  |  |
| A(H1N1)pdm09 | 31/ 407 | 7.6 |  | 366/1767 | 20.7 |  | 68.4 | 55.4 ,78.9 |  | 55.8 | 31.7,72.2 |
| A(H3N2) | 35/ 234 | 15.0 |  | 366 /1767 | 20.7 |  | 32.7 | 3.0,54.5 |  | 44.6 | 13.4, 65.3 |
| B | 44 / 382 | 11.5 |  | 366 /1767 | 20.7 |  | 50.2 | 31.1, 64.8 |  | 43.3 | 15.3,,62.6 |
| Underlying Disease |  |  |  |  |  |  |  |  |  |  |  |
| Yes | 28 / 161 | 17.4 |  | 100/262 | 38.2 |  | 65.9 | 45.6,79.1 |  | 77.5 | 47.6,90.8 |
| No | 81/860 | 9.4 |  | 266/1501 | 17.7 |  | 51.7 | 37.4,63.1 |  | 45.4 | 25.7, 60.2 |
| Exposure to similar symptoms | |  |  |  |  |  |  |  |  |  |  |
| Yes | 54 / 485 | 11.1 |  | 148 / 678 | 21.8 |  | 55.1 | 37.5,68.2 |  | 56.4 | 33.0,72.0 |
| No | 54 / 534 | 10.1 |  | 216 /1083 | 19.9 |  | 54.8 | 38.4, 67.4 |  | 46.5 | 20.5,64.3 |
| Time period |  |  |  |  |  |  |  |  |  |  |  |
| Aug 2009 - Dec 2009 | 5 / 44 | 11.9 |  | 25 / 173 | 14.5 |  | 24.1 | 96.6,76.6 |  | 21.4 | -148.0,78.3 |
| Jan 2010 - May 2010 | 10 / 113 | 8.8 |  | 22 / 151 | 14.6 |  | 43.1 | -22.6,75.2 |  | 47.2 | -22.1, 78.5 |
| May 2010 - Oct 2010 | 30 / 416 | 7.2 |  | 53 / 376 | 14.1 |  | 52.6 | 24.6, 70.7 |  | 43.5 | 2.9,67.5 |
| Nov 2010 to May 2011 | 4 / 22 | 18.2 |  | 52 / 210 | 24.8 |  | 32.5 | -90.8,81.1 |  | 36.5 | -118.0, 85.0 |
| June 2011 - Dec 2011 | 25/175 | 14.3 |  | 107/373 | 28.7 |  | 58.6 | 31.0,74.8 |  | 60.6 | 32.0, 77.8 |
| Jan 2012 - June 2012 | 6 /21 | 28.6 |  | 31 / 120 | 25.8 |  | -14.8 | -210, 61.8 |  | -39.9 | -401.0, 62.7 |
| Jun 2012 - Jan 2013 | 30/232 | 12.9 |  | 76/ 364 | 20.9 |  | 43.7 | 11.8, 64.9 |  | 49.7 | 14.0, 71.2 |

* Adjusted for age using recursive spline and epiweek

** Problems with convergence, failure to converge or perfect separation.
